# Supplementary material for: Circulating KRAS G12D but not G12V is associated with survival in metastatic pancreatic ductal adenocarcinoma
Source: Nat Commun. 2024 Jul 9;15:5763. doi: 10.1038/s41467-024-49915-5 (PMC11233636; doi:10.1038/s41467-024-49915-5)
Supplement: Supplementary file 1 — Supplementary Information [file 41467_2024_49915_MOESM1_ESM.pdf]

Circulating KRAS G12D but not G12V is associated with survival in metastatic pancreatic ductal adenocarcinoma

Supplementary Information

Table of Contents

Supplementary Methods	2
Supplementary Table 1	4
Supplementary Figure 1	5
Supplementary Table 2	6
Supplementary Table 3	7
Supplementary Figure 2	8
Supplementary Figure 4	10
Supplementary Figure 5	11
Supplementary Figure 6	12
Supplementary Table 4	13
Supplementary Figure 7	14
Supplementary Figure 8	15
Supplementary Table 5	16
Supplementary Figure 9	17
Supplementary Figure 10	18
Supplementary Figure 11	19
Supplementary Table 6	20
Supplementary Figure 12	21

Supplementary Methods

For the PRINCE trial, peripheral blood was collected into Streck Cell-Free DNA Blood Collection Tubes tubes (Streck #0230470). For the SOC cohort, specimens were collected in K2EDTA Blood Collection Tubes (EMSCO Fisher #0265732) or Streck Cell-Free DNA Blood Collection tubes. K2EDTA collected specimens were processed to plasma within 3 hours of collection while Streck collected specimens were processed within 7 days. Blood processing occurred at room temperature with an initial centrifugation at 1600 g for 10 minutes (centrifuge brake-off). The upper, plasma, layer was transferred to a new tube and centrifuged again (one or two more times) at 3000 g for 10 minutes (EDTA) or 4122 g for 15 minutes (Streck), again with centrifuge brake-off. The final plasma layer was isolated and banked in 1 mL aliquots in 2mL Sarstedt tubes (Sarstedt #72.694.416) at -80 C.

Circulating cell-free DNA (ccfDNA) extraction using the QIAmp Circulating Nucleic Acid Kit (Qiagen #55114) was performed according to the manufacturer's protocol with two modifications: the proteinase K digestion was extended to 1 hour and final elution was performed twice with 30 µL of Buffer AVE (total 60 µL). Extraction with the QIAmp MinElute ccfDNA Mini Kit (Qiagen #55204*)* was also performed according to the manufacturer's instructions with a single modification: final elution in 30 µL of ultra-clean water run through the column twice. Samples were stored at 4 C prior to quantification.

Quantitative PCR for a 115 bp amplicon of human ALU repeat element was used to determine ccfDNA concentration (primers: forward 5'-CCTGAGGTCAGGAGTTCGAG-3' and reverse 5'-CCCGAGTAGCTGGGATTACA-3')[1]. Samples were diluted 1:10 with nuclease free water and a standard curve was generated by serial dilution of a commercial DNA standard (Promega G3041). Power SYBR Green PCR Master Mix (Applied Biosystems #4367659) was used according to the manufacturer's instructions on a ViiA 7 Real-Time PCR System (Applied Biosystems). Results were analyzed using QuantStudio Real-Time PCR Software (Applied Biosystems).

Prior to droplet digital PCR (ddPCR), ccfDNA was pre-amplified for the *KRAS* G12 locus (primers: forward 5'- AGGCCTGCTGAAAATGACTGAATAT-3' and reverse 5'-GCTGTATCGTCAAGGCACTCTT-3'). This PCR was performed using the Q5 Hot Start Hi-Fidelity Master Mix (NEB #M0494), and 0.05 µM primers with either 15 µl of QIAmp Circulating Nucleic Acid Kit ccfDNA or up to 24 µl of QIAmp MinElute ccfDNA Mini Kit ccfDNA (with a maximum of 30 ng). PCR was performed in the Veriti 96 Well Thermal Cycler (Applied Biosystems) with the following program: 98 C for 3 minutes, 9 cycles of 98 C for 10 seconds, 63 C 3 minutes, and 72 C 30 seconds, followed by 72 C for 2 minutes[2]. Pre-amplified material was diluted 1:4 with TE buffer and stored at 4 C in the short-term and -20 C in the long-term storage.

For RainDrop ddPCR, initial characterization was carried out with a multiplex reaction (using the same primers as pre-amplification above) and probes for *KRAS* G12WT (VIC-TTGGAGCTGGTGGCGT-MGBNFQ), G12D (FAM-TGGAGCTGATGGCGT-MGBNFQ), G12V (FAM-GAGCTGTTGGCGT-MGBNFQ), and G12R (FAM-TTGGAGCTCGTGGCGT-MGBNFQ). Reactions contained 2x TaqMan Genotyping Master Mix (Applied Biosystems #4371355), 25x Droplet Stabilizer (RainDance #30-07026), 200nM primers, G12WT, G12D, and G12V probes at 100 nM, G12R probe at 50 nM and 10 µl of pre-amplified ccfDNA in a total of 30 µl. Final quantification of variant allele fraction (VAF) was carried out in a duplex reaction with the above conditions but only two probes (G12WT and G12Mutant of interest) at 100 nM each. Droplets were generated from 25 µl of reaction mix on the RainDrop Source instrument (RainDance Technologies, Inc.). Next, PCR was performed in a GeneTouch PCR Thermal Cycler (Bioer Technology Co., Ltd.); 0.6 C per second ramp rate with the following cycling conditions, 95 C for 10 minutes, 45 cycles of 95 C for 15

seconds and 60 C for 1 minute, 10 minutes at 5 C, and held at 4 C. Droplets were read on the RainDrop Sense instrument (RainDance Technologies, Inc.) and data analyzed in RainDrop Analyst software (RainDance Technologies, Inc.). Droplet counts were adjusted to copy counts using the Poison correction and background adjusted based on 20 healthy control samples[3].

For QX200 platform (Bio-Rad Laboratories, Inc) ddPCR, initial characterization with *KRAS G12/G13* Screening Kit (Bio-Rad, #1863506) was carried out according to manufacturer's instruction utilizing the maximum amount of pre-amplified material that resulted in less than 100,000 copies measured (to not overload the assay). Subsequent analysis with individual variant assays were carried out similarly (G12A, dHsaMDV2510586; G12C, dHsaMDV2510584; G12D, dHsaMDV2510596; G12R, dHsaMDV2510590; G12S, dHsaMDV2510588; G12V, dHsaMDV2510592; G13D, dHsaMDV2510598).

<u>Supplementary Methods References</u>

1. Fawzy, A., Sweify, K.M., El-Fayoumy, H.M. & Nofal, N. Quantitative analysis of plasma cell-free DNA and its DNA integrity in patients with metastatic prostate cancer using ALU sequence. *J Egypt Natl Canc Inst* **28**, 235-242 (2016).
2. Jackson, J.B.*, et al.* Multiplex Preamplification of Serum DNA to Facilitate Reliable Detection of Extremely Rare Cancer Mutations in Circulating DNA by Digital PCR. *J Mol Diagn* **18**, 235-243 (2016).
3. Milbury, C.A.*, et al.* Determining lower limits of detection of digital PCR assays for cancer-related gene mutations. *Biomol Detect Quantif* **1**, 8-22 (2014).

Patient#	Prior Treatment	Tissue KRAS Variant	Baseline Plasma ctKRAS Variant
PRINCE001	Yes	No Sample	No Sample
PRINCE002	Yes	Q61H	No Sample
PRINCE003	Yes	G12D	G12D
PRINCE004	No	G12R	No Sample
PRINCE005	No	G12V	Inconclusive
PRINCE006	Yes	G12D	G12D
PRINCE007	No	No Sample	Inconclusive
PRINCE008	Yes	G12V	G12V
PRINCE009	Yes	G12V	G12V
PRINCE010	No	G12V	G12V
PRINCE011	Yes	Not Detected	Inconclusive
PRINCE012	Yes	G12V	G12V
PRINCE013	No	No Sample	G12D
PRINCE014	Yes	G12V	G12V
PRINCE015	No	G12D	G12D
PRINCE016	No	G12D	G12D
PRINCE017	No	No Sample	G12D
PRINCE018	No	G12D	G12D
PRINCE019	Yes	No Sample	G12D
PRINCE020	No	G12D	G12D
PRINCE021	Yes	G12D	Inconclusive
PRINCE022	No	G12V	G12V
PRINCE023	No	Not Detected	No Sample
PRINCE024	Yes	No Sample	Inconclusive
PRINCE025	No	G12V	G12V
PRINCE026	No	G12D	Inconclusive
PRINCE027	No	G12V	G12V
PRINCE028	Yes	Not Detected	Conclusive Negative
PRINCE029	Yes	G12D	Inconclusive
PRINCE030	No	Not Detected	Inconclusive
PRINCE031	No	No Sample	G12V
PRINCE032	No	G12D	G12D
PRINCE033	Yes	G12R	Inconclusive
PRINCE034	No	Not Detected	Inconclusive
PRINCE035	No	G12R	G12R
PRINCE036	No	G12V	G12V
PRINCE037	No	No Sample	G12D
PRINCE038	No	G12V	G12V
PRINCE039	No	G12D	G12D
PRINCE040	No	G12R	G12R
PRINCE041	Yes	G12D	G12D
PRINCE042	No	G12D	G12D
PRINCE043	No	G12D	No Sample
PRINCE044	No	G12D	G12D
PRINCE045	No	Not Detected	G12V
PRINCE046	No	Not Detected	No Sample
PRINCE047	No	G12R	G12R
PRINCE048	No	G12V	G12V
PRINCE049	No	Not Detected	Inconclusive
PRINCE050	No	No Sample	G12D
PRINCE051	No	G12D	G12D
PRINCE052	No	G12V	G12V
PRINCE053	No	No Sample	G12D
PRINCE054	No	G12D	G12D
PRINCE055	No	G12V	G12V
PRINCE056	No	G12V	G12V
PRINCE057	No	No Sample	G12D
PRINCE058	No	G12V	G12V
PRINCE059	Yes	No Sample	G12D
PRINCE060	No	No Sample	No Sample
PRINCE061	No	G12D	G12D
PRINCE062	No	No Sample	G12R
PRINCE063	Yes	Not Detected	Inconclusive
PRINCE064	Yes	G12D	G12D
PRINCE065	No	Not Detected	Inconclusive
PRINCE066	No	G12V	Inconclusive
PRINCE067	Yes	No Sample	G12D
PRINCE068	No	G12V	G12V
PRINCE069	Yes	G12V	G12V
PRINCE070	No	No Sample	G12D
PRINCE071	Yes	Not Detected	No Sample
PRINCE072	No	G12D	G12D
PRINCE073	No	G12R	G12R
PRINCE074	Yes	No Sample	Inconclusive
PRINCE075	No	Q61H	G12S
PRINCE076	Yes	Not Detected	Inconclusive
PRINCE077	Yes	No Sample	G12D
PRINCE078	No	No Sample	G12D
PRINCE079	Yes	G12D	G12D
PRINCE080	Yes	G12D	G12D
PRINCE081	No	Not Detected	G12D
PRINCE082	No	G12D	G12D
PRINCE083	No	Not Detected	Conclusive Negative
PRINCE084	No	No Sample	No Sample
PRINCE085	No	Not Detected	G12D
PRINCE086	No	No Sample	Conclusive Negative
PRINCE087	No	No Sample	G12V
PRINCE088	No	G12R	G12R
PRINCE089	No	Not Detected	Inconclusive
PRINCE090	No	Q61H	Inconclusive
PRINCE091	No	G12V	G12V
PRINCE092	No	G12D	G12D
PRINCE093	No	No Sample	No Sample
PRINCE094	No	No Sample	G12D
PRINCE095	No	Not Detected	No Sample
PRINCE096	No	No Sample	Inconclusive
PRINCE097	No	No Sample	G12D
PRINCE098	No	G12V	No Sample
PRINCE099	No	G12D	G12D
PRINCE100	No	No Sample	G12V
PRINCE101	No	G12V	Inconclusive
PRINCE102	No	Not Detected	G12R
PRINCE103	No	No Sample	No Sample
PRINCE104	No	G12D	G12D
PRINCE105	No	No Sample	No Sample
PRINCE106	No	G12D	G12D+G12V
PRINCE107	Yes	No Sample	G12D+G12V
PRINCE108	No	No Sample	G12V
PRINCE109	No	G12V	G12V
PRINCE110	No	G12D	G12D
PRINCE111	No	No Sample	G12D
PRINCE112	No	No Sample	G12V
PRINCE113	No	No Sample	G12D+G12V
PRINCE114	No	Not Detected	G12D
PRINCE115	No	No Sample	G12V
PRINCE116	No	G12V	G12V
PRINCE117	No	Not Detected	G12V
PRINCE118	No	No Sample	G12D+G12V
PRINCE119	No	No Sample	G12D
PRINCE120	Yes	G12R	Inconclusive
PRINCE121	Yes	Not Detected	G12V
PRINCE122	No	No Sample	Inconclusive
PRINCE123	Yes	Q61H	Inconclusive
PRINCE124	No	No Sample	Inconclusive
PRINCE125	Yes	Q61R	Inconclusive
PRINCE126	No	G12D	G12D
PRINCE127	Yes	G12D	G12D
PRINCE128	Yes	G12V	G12V
PRINCE129	Yes	Not Detected	Inconclusive

Supplementary Table 1. Individual PRINCE trial cohort patient data regarding prior treatment history and KRAS variant detection in tissue and baseline plasma.

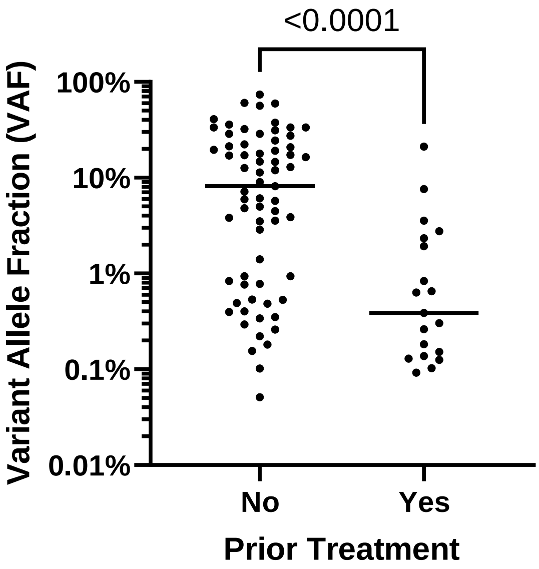

Supplementary Figure 1. Prior Treatment and ctKRAS VAF Levels for PRINCE patients. Among 86 PRINCE trial patients with a baseline plasma ctKRAS mutation detected, VAF levels were significantly higher for patients without prior treatment (n=67) than for those with prior treatment (n=19; Mann-Whitney test, (two-sided). Source data are provided as a Source Data file.

	PRINCE (N=83)	SOC (N=85)	P-value
Age			
Median (Range)	62 (35-79)	67 (38-87)	0.0318*
<65	46 (55%)	38 (45%)	0.2169†
>65	37 (45%)	47 (55%)	
Sex			
Female	35 (42%)	37 (44%)	0.8773†
Male	48 (58%)	48 (56%)	
Race			
Asian	6 (7%)	1 (1%)	0.1089‡
Black or African American	3 (4%)	5 (6%)	
Caucasian	72 (87%)	73 (86%)	
Other	2 (2%)	6 (7%)	
Ethnicity			
Hispanic or Latino	1 (1%)	0 (0%)	0.5529†
Not Hispanic or Latino	82 (99%)	85 (100%)	
ECOG PS			
0	36 (43%)	29 (34%)	0.0011‡
1	47 (57%)	41 (48%)	
2	0 (0%)	13 (15%)	
3	0 (0%)	2 (2%)	
ctKRAS Variant			
G12D	33 (40%)	34 (40%)	0.6149‡
G12V	23 (28%)	20 (24%)	
G12R	7 (8%)	13 (15%)	
Other	4 (5%)	2 (2%)	
Negative	16 (19%)	16 (19%)	

***Mann-Whitney Test (two-sided), †Fisher's Exact Test (two-sided), ‡Chi-square Test (two-sided)**

Supplementary Table 2. Characteristics of patients with baseline plasma analyzed in the PRINCE and standard of care (SOC) cohorts. ECOG PS refers to Eastern Cooperative Oncology Group Performance Status scale. All PRINCE patients received chemoimmunotherapy as part of a clinical trial. Among the 85 SOC patients, 36 received gemcitabine-based therapy (including gemcitabine alone, gemcitabine/nab-paclitaxel, and gemcitabine/cisplatin) and 49 patients received folfirinox-based therapy (including folfirinox, FOLFOX, mFolfirinox, mFOLFOX, and mFOLFOX 6). Source data are provided as a Source Data file.

PRINCE patients

	G12D Bearing tumor (N=33)	G12V Bearing tumor (N=23)	P-value
Age			
Median (Range)	62 (44-77)	63 (43-79)	0.8395*
<65	20 (61%)	12 (52%)	0.5903†
>65	13 (39%)	11 (48%)	
Sex			
Female	18 (55%)	10 (43%)	0.5875†
Male	15 (45%)	13 (57%)	
Race			
Asian	2 (6%)	3 (13%)	0.6511‡
Black or African American	2 (6%)	1 (4%)	
Caucasian	29 (88%)	19 (83%)	
Other	0 (0%)	0 (0%)	
Ethnicity			
Hispanic or Latino	0 (0%)	1 (4%)	0.4107†
Not Hispanic or Latino	33 (100%)	22 (96%)	
ECOG PS			
0	15 (45%)	10 (43%)	>0.9999†
1	18 (55%)	13 (57%)	
2	0 (0%)	0 (0%)	
3	0 (0%)	0 (0%)	

***Mann-Whitney Test (two-sided), †Fisher's Exact Test (two-sided), ‡Chi-square Test (two-sided)**

SOC patients

	G12D Bearing tumor (N=34)	G12V Bearing tumor (N=20)	P-value
Age			
Median (Range)	65 (38-87)	64 (46-80)	0.5051*
<65	17 (50%)	10 (50%)	>0.9999†
>65	17 (50%)	10 (50%)	
Sex			
Female	13 (38%)	10 (50%)	0.5694†
Male	21 (62%)	10 (50%)	
Race			
Asian	0 (0%)	0 (0%)	0.6426‡
Black or African American	2 (6%)	2 (10%)	
Caucasian	29 (85%)	15 (75%)	
Other	3 (9%)	3 (15%)	
Ethnicity			
Hispanic or Latino	0 (0%)	0 (0%)	>0.9999†
Not Hispanic or Latino	34 (100%)	20 (100%)	
ECOG PS			
0	9 (26%)	2 (10%)	0.3448‡
1	18 (53%)	15 (75%)	
2	6 (18%)	3 (15%)	
3	1 (3%)	0 (0%)	

***Mann-Whitney Test (two-sided), †Fisher's Exact Test (two-sided), ‡Chi-square Test (two-sided)**

Supplementary Table 3. Of 67 therapy-naïve PRINCE patients (left) who had a *KRAS* variant detected in tissue or plasma, 33 had a *KRAS* G12D and 23 had G12V. Of 54 therapy-naïve SOC patients (right) who had a baseline *KRAS* variant, 34 had *KRAS* G12D and 20 had G12V. All patients shown in these tables are therapy-naïve. ECOG PS refers to Eastern Cooperative Oncology Group Performance Status scale. Source data are provided as a Source Data file.

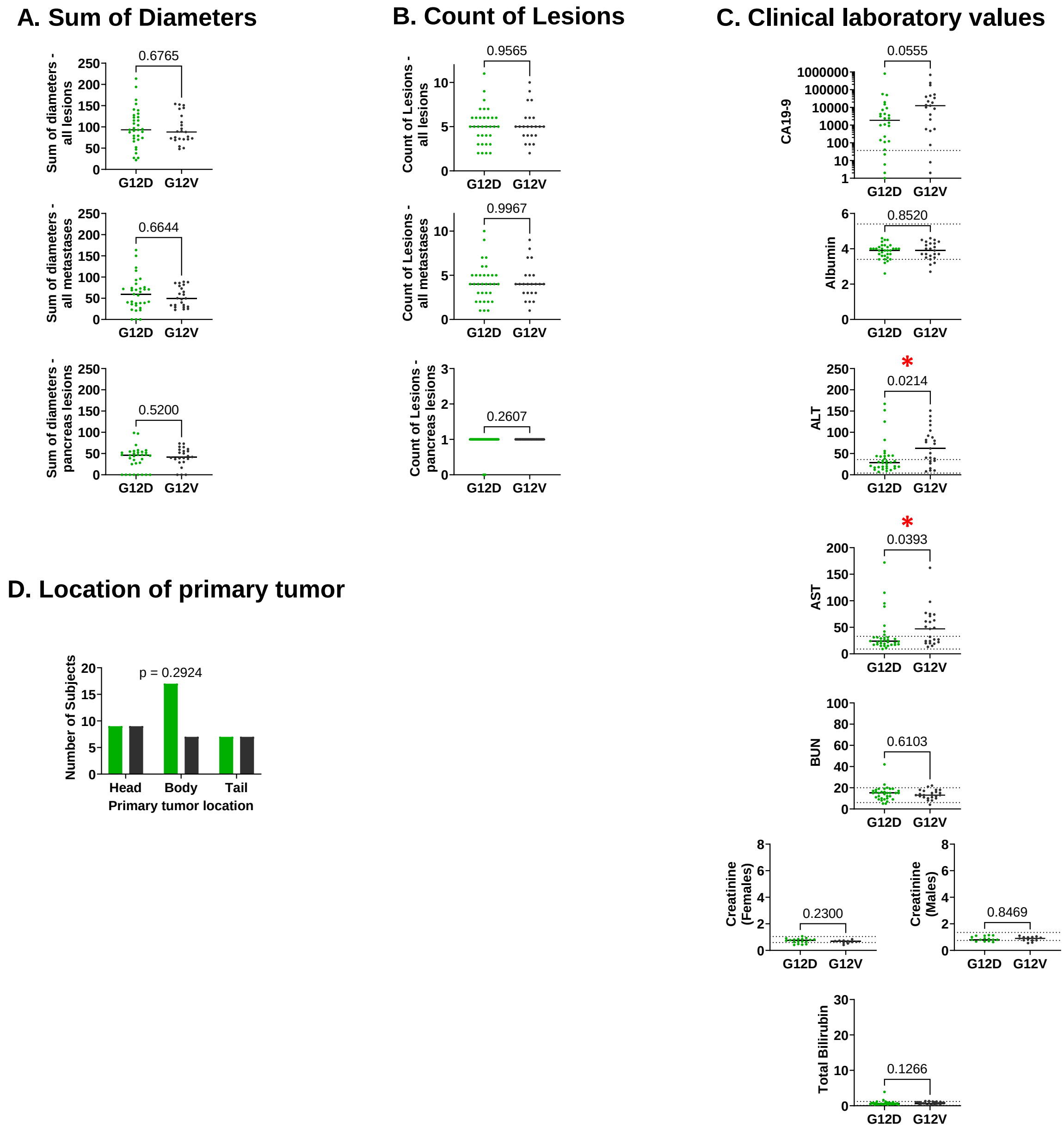

Supplementary Figure 2. Comparison of clinical variable values for G12D- vs G12V-bearing tumors for therapy-naïve patients in PRINCE cohort. Shown is analysis of 33 patients with a G12D-bearing tumor and 23 patients with G12V for all variables except CA19-9, for which there were 26 patients with G12D and 21 patients with G12V. A red asterisk * indicates significance at 0.05. Mann-Whitney test (two-sided) used for all variables other than location of primary tumor, Chi-square test (two-sided). Dotted horizontal lines for clinical laboratory values shown in C indicate upper and lower limits of normal clinical values. Green denotes ctKRAS G12D and dark grey is G12V. Abbreviation ALT (Alanine Transaminase), AST (Aspartate Transaminase), and BUN (Blood Urea Nitrogen) are used. Source data are provided as a Source Data file.

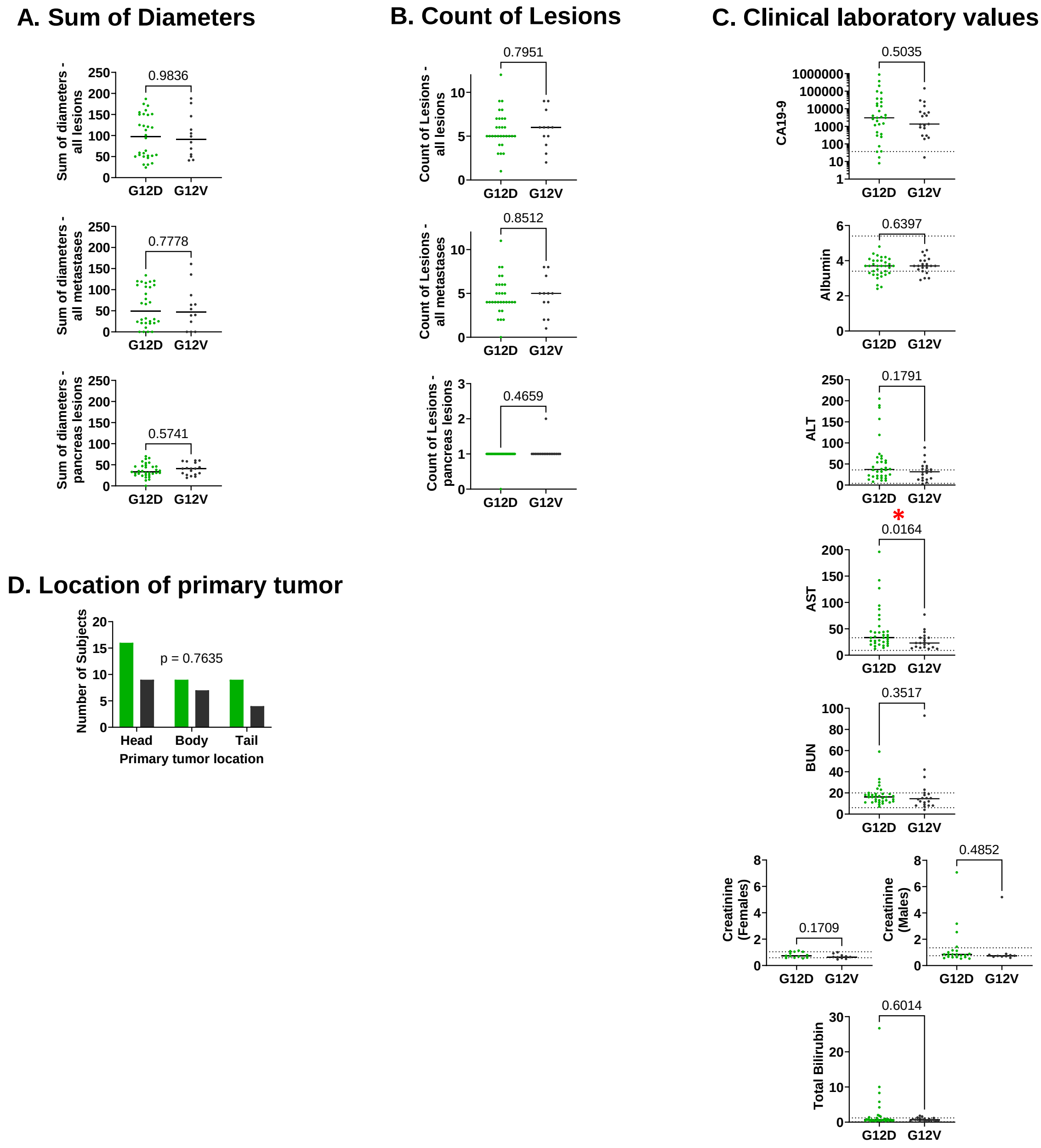

Supplementary Figure 3. Comparison of clinical variable values for G12D- vs G12V-bearing tumors for patients in SOC cohort. Not all variables could be collected for all patients. For imaging analysis shown in A and B, measures of pancreatic lesions are shown for 33 patients with a G12D-bearing tumor and for 18 with G12V, all other imaging variables were analyzed for 30 patients with a G12D-bearing tumor and for 12 with G12V. Shown in C, D, and E is analysis of CA19-9 is shown for 31 G12D patients and 19 G12V patients, the remaining variables are shown for 34 patients with a G12D-bearing tumor and for 20 patients with G12V. Mann-Whitney test (two-sided) used for all variables other than location of primary tumor, for which Chi-square test (two-sided) was used. Dotted horizontal lines for clinical laboratory values shown in C indicate upper and lower limits of normal clinical values. Abbreviation ALT (Alanine Transaminase), AST (Aspartate Transaminase), and BUN (Blood Urea Nitrogen) are used. Green denotes ctKRAS G12D and dark grey is G12V. Source data are provided as a Source Data file.

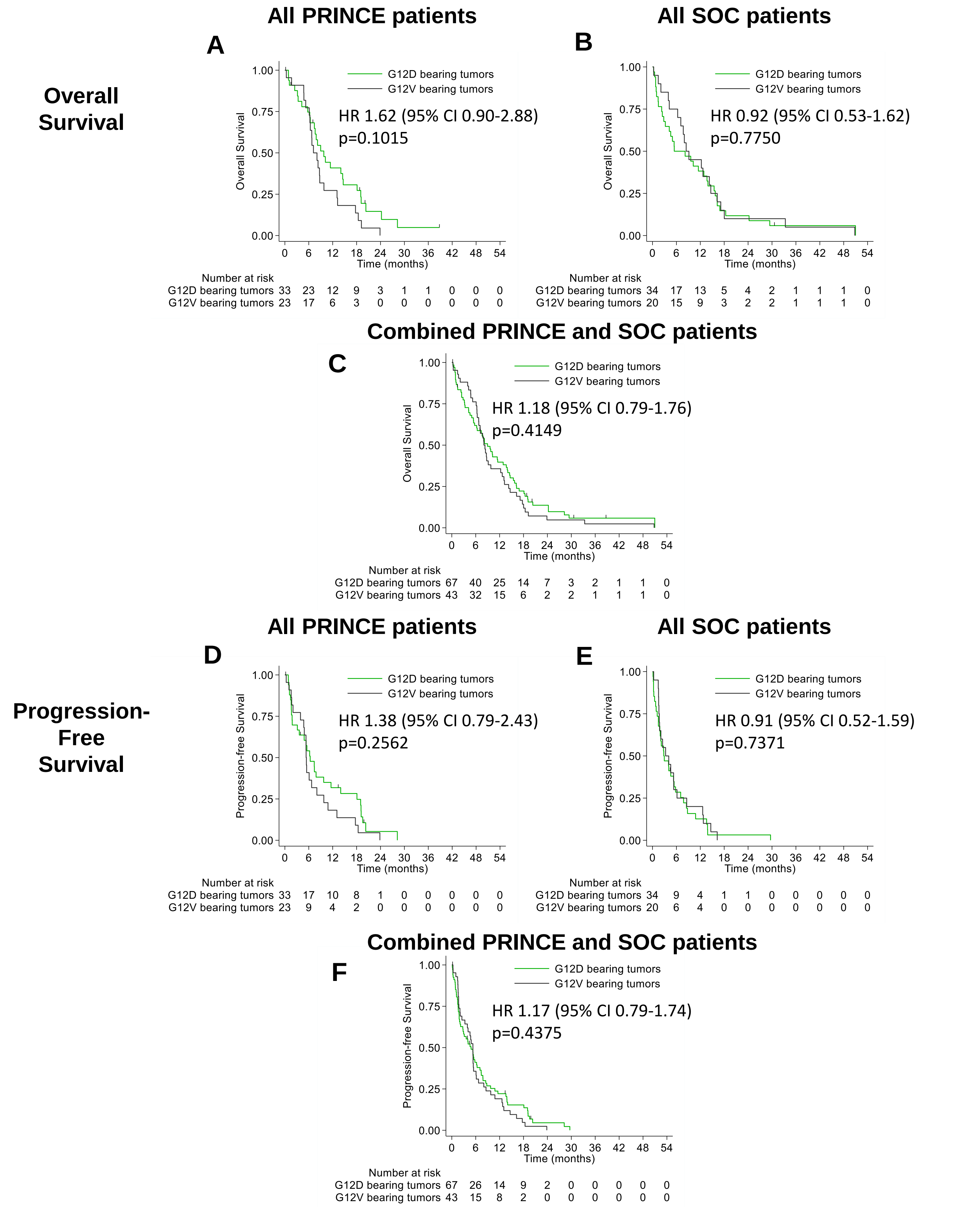

Supplementary Figure 4. Association of tumor *KRAS* mutation status (G12D- vs G12V-bearing tumors) with overall survival (A-C) and progression-free survival (D-F) for therapy-naive patients enrolled in PRINCE trial or who received standard of care (SOC) therapy, or the pooled therapy-naïve PRINCE + SOC cohorts. Cox regression hazard ratios (HR) and 95% confidence intervals (CI) are shown with log-rank p-values. Source data are provided as a Source Data file.

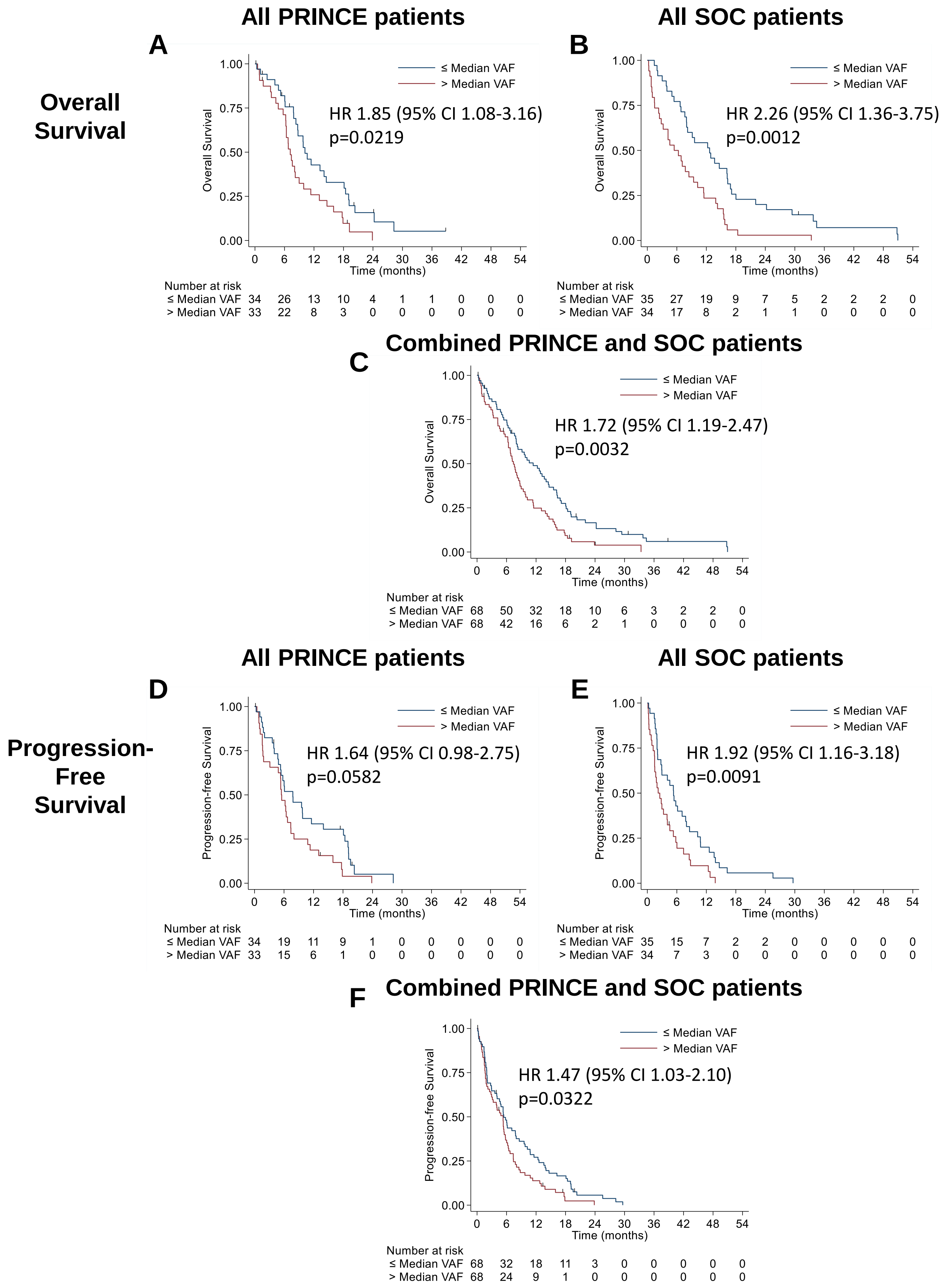

Supplementary Figure 5. Baseline plasma ctKRAS variant allele fraction (VAF) dichotomized at the median and associated with survival for therapy-naïve PRINCE patients (A and D, n=67), SOC patients (B and E, n=69) or combined therapy naïve PRINCE and SOC patients (C and F, N =136) with any ctKRAS variant. Overall survival shown in A, B and C, progression-free survival shown in D, E, and F. Cox regression hazard ratios (HR) and 95% confidence intervals (CI) are shown with log-rank p-values. Source data are provided as a Source Data file.

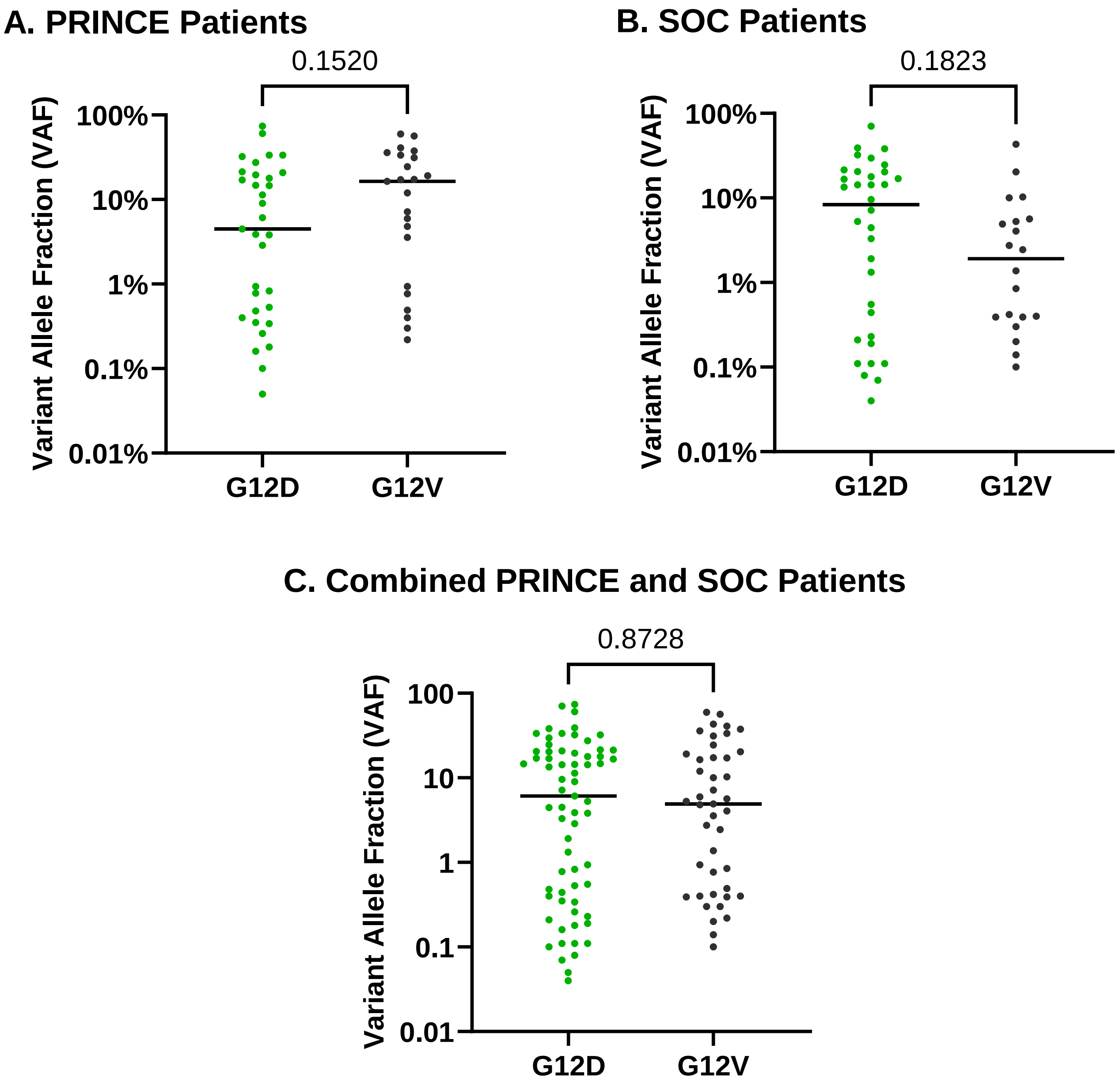

Supplementary Figure 6. VAF levels do not differ significantly in therapy-naïve patients between ctKRAS G12D and ctKRAS G12V (Mann-Whitney test, two-sided) in the A) PRINCE (n=33, and 23, respectively) or B) standard of care (SOC, n=34, and 20, respectively) cohorts. Shown in C) is the pooled therapy-naïve PRINCE and SOC cohort. (n=67, and 43, respectively). Green denotes ctKRAS G12D and dark gray is G12V. Source data are provided as a Source Data file.

		PRINCE Patients							
		G12D (n=33)				G12V (n=23)			
		Univariate		Multivariate		Univariate		Multivariate	
		HR [95% CI]	p-value	HR [95% CI]	p-value	HR [95% CI]	p-value	HR [95% CI]	p-value
OS	logVAF	**1.59 [1.01-2.51]**	**0.046**	**1.98 [1.10-3.56]**	**0.022**	0.92 [0.54-1.57]	0.765	1.10 [0.57-2.11]	0.781
	Age	0.97 [0.92-1.01]	0.145	0.98 [0.94-1.03]	0.534	1.05 [1.00-1.11]	0.072	1.03 [0.96-1.10]	0.404
	Sex	1.95 [0.89-4.27]	0.093	2.42 [0.99-5.93]	0.053	0.38 [0.14-1.02]	0.056	0.51 [0.14-1.78]	0.290
	ECOG PS[1]	1.33 [0.61-2.90]	0.481	1.21 [0.55-2.68]	0.631	1.71 [0.66-4.41]	0.270	1.67 [0.57-4.95]	0.352
	logSOD[2]	1.43 [0.27-7.70]	0.676	0.23 [0.03-1.94]	0.176	1.12 [0.03-41.68]	0.952	0.74 [0.01-56.54]	0.891
PFS	logVAF	**1.74 [1.11-2.73]**	**0.017**	**2.14 [1.22-3.76]**	**0.008**	1.00 [0.61-1.64]	0.991	1.24 [0.68-2.26]	0.489
	Age	0.95 [0.91-1.00]	0.058	0.97 [0.92-1.02]	0.245	1.05 [0.99-1.12]	0.081	1.03 [0.95-1.10]	0.492
	Sex	2.01 [0.95-4.26]	0.069	**2.40 [1.02-5.62]**	**0.045**	0.46 [0.18-1.13]	0.090	0.52 [0.16-1.62]	0.258
	ECOG PS[1]	1.14 [0.55-2.39]	0.723	1.08 [0.51-2.30]	0.842	1.75 [0.70-4.37]	0.232	1.80 [0.60-5.36]	0.294
	logSOD[2]	1.66 [0.31-8.80]	0.552	0.21 [0.03-1.63]	0.135	1.39 [0.04-49.62]	0.858	0.76 [0.02-30.28]	0.884

		SOC Patients							
		G12D (n=31)				G12V (n=19)			
		Univariate		Multivariate		Univariate		Multivariate	
		HR [95% CI]	p-value	HR [95% CI]	p-value	HR [95% CI]	p-value	HR [95% CI]	p-value
OS	logVAF	**2.03 [1.31-3.15]**	**0.001**	**2.43 [1.42-4.16]**	**0.001**	1.41 [0.70-2.86]	0.336	2.08 [0.92-4.71]	0.078
	Age	1.02 [0.98-1.06]	0.243	**1.06 [1.01-1.11]**	**0.011**	1.00 [0.95-1.06]	0.855	1.01 [0.94-1.08]	0.876
	Sex	1.62 [0.75-3.50]	0.220	**3.09 [1.28-7.45]**	**0.012**	0.60 [0.23-1.58]	0.299	0.33 [0.08-1.41]	0.134
	ECOG PS[1]	**2.11 [1.30-3.43]**	**0.003**	**2.43 [1.33-4.44]**	**0.004**	**3.16 [1.12-8.87]**	**0.029**	**4.26 [1.12-16.18]**	**0.033**
	logCA19-9	1.27 [0.94-1.72]	0.114	0.88 [0.63-1.23]	0.458	0.86 [0.44-1.68]	0.656	1.36 [0.52-3.54]	0.532
PFS	logVAF	**1.58 [1.08-2.32]**	**0.020**	**1.73 [1.14-2.63]**	**0.010**	1.90 [0.99-3.64]	0.054	1.64 [0.80-3.38]	0.179
	Age	1.03 [0.99-1.07]	0.154	**1.05 [1.01-1.10]**	**0.025**	0.97 [0.92-1.03]	0.336	0.97 [0.90-1.05]	0.414
	Sex	2.00 [0.86-4.65]	0.106	**3.18 [1.22-8.28]**	**0.018**	1.30 [0.51-3.34]	0.581	1.71 [0.46-6.42]	0.427
	ECOG PS[1]	1.46 [0.93-2.30]	0.097	1.28 [0.78-2.11]	0.327	2.48 [0.57-10.79]	0.226	2.51 [0.50-12.55]	0.261
	logCA19-9	1.13 [0.82-1.56]	0.439	0.89 [0.63-1.28]	0.534	1.01 [0.53-1.93]	0.981	0.78 [0.33-1.83]	0.570

		Combined PRINCE and SOC Patients							
		G12D (n=53)				G12V (n=32)			
		Univariate		Multivariate		Univariate		Multivariate	
		HR [95% CI]	p-value	HR [95% CI]	p-value	HR [95% CI]	p-value	HR [95% CI]	p-value
OS	logVAF	**1.77 [1.27-2.48]**	**0.001**	**2.27 [1.44-3.60]**	**0.0002**	1.06 [0.66-1.69]	0.810	1.41 [0.79-2.53]	0.245
	Age	1.00 [0.97-1.03]	0.998	1.03 [0.99-1.07]	0.116	1.05 [1.00-1.09]	0.053	1.03 [0.97-1.09]	0.323
	Sex	**1.97 [1.08-3.57]**	**0.026**	**3.31 [1.53-7.17]**	**0.002**	**0.42 [0.20-0.92]**	**0.029**	0.50 [0.20-1.24]	0.135
	ECOG PS[1]	**1.92 [1.23-2.97]**	**0.004**	**1.70 [1.05-2.74]**	**0.030**	**1.89 [1.00-3.54]**	**0.049**	2.28 [0.98-5.30]	0.055
	logSOD[2]	**3.44 [1.07-11.04]**	**0.038**	0.43 [0.09-2.13]	0.303	2.95 [0.23-37.35]	0.404	0.23 [0.01-5.30]	0.355
	logCA19-9	1.21 [0.97-1.52]	0.092	1.04 [0.84-1.29]	0.717	0.80 [0.57-1.13]	0.206	0.92 [0.61-1.39]	0.690
PFS	logVAF	**1.65 [1.18-2.30]**	**0.003**	**2.05 [1.35-3.10]**	**0.001**	0.96 [0.64-1.45]	0.854	1.25 [0.77-2.03]	0.357
	Age	1.00 [0.97-1.03]	0.920	1.02 [0.99-1.06]	0.253	1.05 [1.00-1.10]	0.069	1.03 [0.98-1.09]	0.274
	Sex	**2.30 [1.26-4.19]**	**0.006**	**3.27 [1.58-6.78]**	**0.001**	0.59 [0.28-1.24]	0.165	1.10 [0.42-2.88]	0.847
	ECOG PS[1]	1.40 [0.92-2.14]	0.120	1.25 [0.78-2.00]	0.346	**2.17 [1.05-4.49]**	**0.037**	1.98 [0.89-4.41]	0.096
	logSOD[2]	2.50 [0.81-7.77]	0.112	0.45 [0.10-1.95]	0.285	2.42 [0.18-32.77]	0.506	1.02 [0.06-17.89]	0.992
	logCA19-9	1.15 [0.92-1.42]	0.218	1.00 [0.81-1.24]	0.972	**0.70 [0.50-0.98]**	**0.038**	0.66 [0.42-1.04]	0.071

[1]ECOG PS = Eastern Cooperative Oncology Group Performance Status

[2]SOD = Sum of diameters for all target lesions

Supplementary Table 4. Cox analysis for baseline plasma log ctKRAS variant allele fraction (VAF) as a continuous variable for therapy-naïve PRINCE (top table), standard of care (SOC, middle table), and combined therapy-naïve PRINCE and SOC (bottom table), with results for ctKRAS G12D on left and for G12V on right. CA19-9 excluded from PRINCE and sum of diameters (SOD) excluded from SOC cohort due to incomplete data. Of the 33 and 31 patients analyzed in the PRINCE and SOC tables respectively for G12D, only the 53 patients with both CA19-9 and SOD values available were included in combined cohort (bottom table). Significant values (without adjustment for multiple testing) indicated by bolded red text. Source data are provided as a Source Data file.

Combined PRINCE and SOC patients

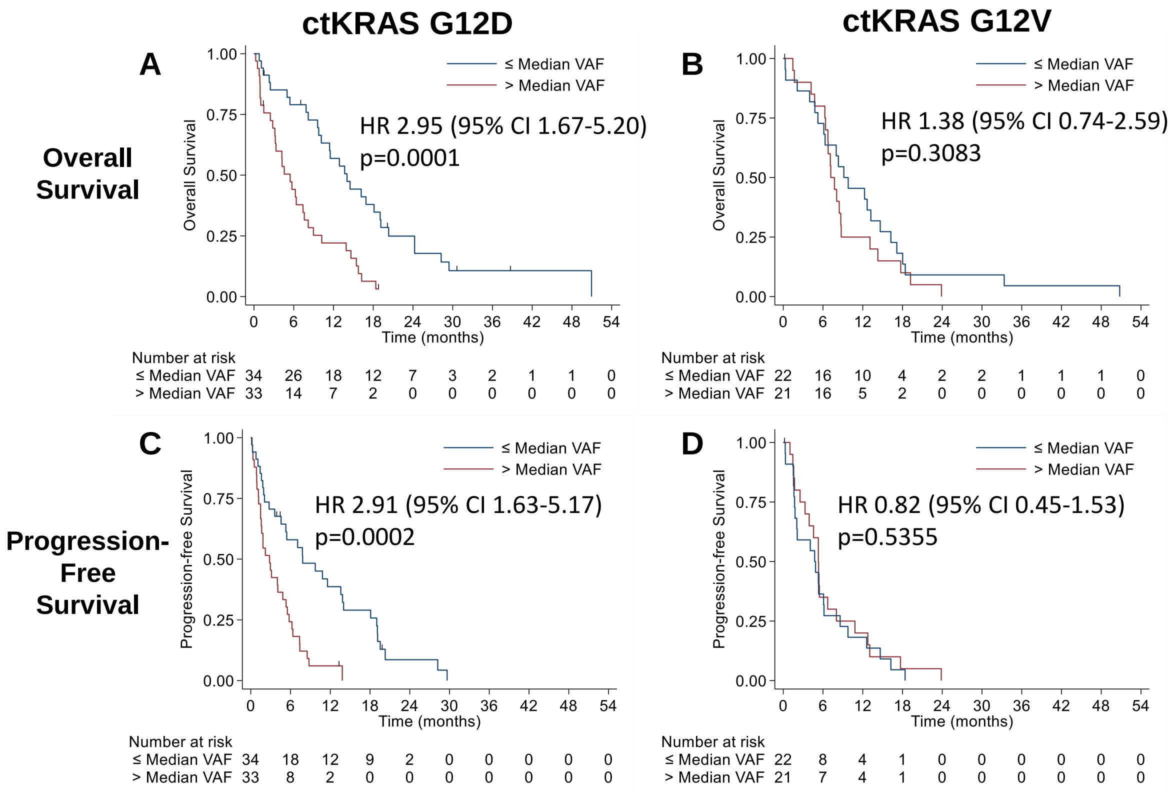

Supplementary Figure 7. Survival association for baseline ctKRAS variant allele fraction (VAF) by variant for therapy-naïve combined PRINCE and standard of care(SOC) patients. Shown are the Kaplan-Meier curves for baseline VAF dichotomized at the median for overall survival (top, A and B) and progression-free survival (bottom, C and D) for patients with G12D- (left, A and C) or G12V-bearing tumors (right, B and D). Cox regression hazard ratios (HR) and 95% confidence intervals (CI) are shown with log-rank p-values. Source data are provided as a Source Data file.

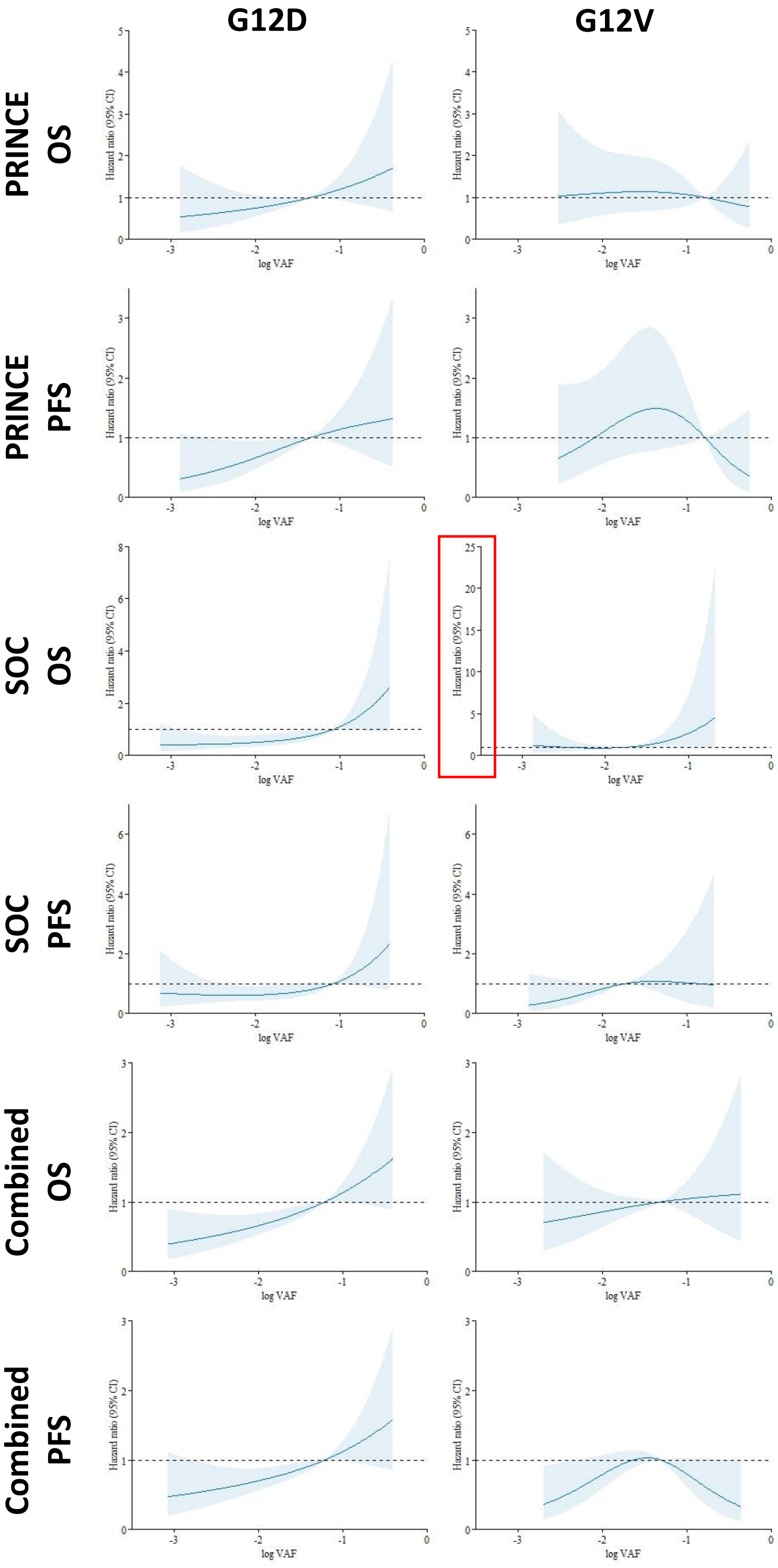

Supplementary Figure 8. Estimated cubic spline functions relating log ctKRAS variant allele fraction (VAF) to results of the Cox models. Results for overall survival (OS) and progression-free survival (PFS) shown for PRINCE (top two rows), standard of care (SOC, middle two rows), and combined PRINCE and SOC patients (bottom two rows). The left column shows results for ctKRAS G12D only, and the right column for ctKRAS G12V only. Red box indicates y-axis maximum for G12V is different than for G12D for the SOC Overall Survival functions. Source data are provided as a Source Data file.

Survival	LoD	G12D	G12V
OS	0.04%	**HR 2.95 (95% CI 1.67-5.20) p=0.0001**	HR 1.38 (95% CI 0.74-2.59) p=0.3083
	0.10%	**HR 2.62 (95% CI 1.47-4.66) p=0.0007**	HR 1.38 (95% CI 0.74-2.59) p=0.3083
	0.25%	**HR 1.98 (95% CI 1.08-3.62) p=0.0244**	HR 1.31 (95% CI 0.68-2.53) p=0.4203
PFS	0.04%	**HR 2.91 (95% CI 1.63-5.17) p=0.0002**	HR 0.82 (95% CI 0.45-1.53) p=0.5355
	0.10%	**HR 2.86 (95% CI 1.58-5.20) p=0.0003**	HR 0.82 (95% CI 0.45-1.53) p=0.5355
	0.25%	HR 1.80 (95% CI 0.97-3.34) p=0.058	HR 0.66 (95% CI 0.34-1.28) p=0.209

LoD = 0.04%		G12D N=53				G12V N=32			
		Univariate		Multivariate		Univariate		Multivariate	
	Variable	HR [95% CI]	p-value	HR [95% CI]	p-value	HR [95% CI]	p-value	HR [95% CI]	p-value
OS	logVAF	**1.77 [1.27-2.48]**	**0.001**	**2.27 [1.44-3.60]**	**0.000**	1.06 [0.66-1.69]	0.810	1.41 [0.79-2.53]	0.245
	Age	1.00 [0.97-1.03]	0.998	1.03 [0.99-1.07]	0.116	1.05 [1.00-1.09]	0.053	1.03 [0.97-1.09]	0.323
	Sex	**1.97 [1.08-3.57]**	**0.026**	**3.31 [1.53-7.17]**	**0.002**	**0.42 [0.20-0.92]**	**0.029**	0.50 [0.20-1.24]	0.135
	ECOGPS[1]	**1.92 [1.23-2.97]**	**0.004**	**1.70 [1.05-2.74]**	**0.030**	**1.89 [1.00-3.54]**	**0.049**	2.28 [0.98-5.30]	0.055
	logSOD[2]	**3.44 [1.07-11.04]**	**0.038**	0.43 [0.09-2.13]	0.303	2.95 [0.23-37.35]	0.404	0.23 [0.01-5.30]	0.355
	logCA19-9	1.21 [0.97-1.52]	0.092	1.04 [0.84-1.29]	0.717	0.80 [0.57-1.13]	0.206	0.92 [0.61-1.39]	0.690
PFS	logVAF	**1.65 [1.18-2.30]**	**0.003**	**2.05 [1.35-3.10]**	**0.001**	0.96 [0.64-1.45]	0.854	1.25 [0.77-2.03]	0.357
	Age	1.00 [0.97-1.03]	0.920	1.02 [0.99-1.06]	0.253	1.05 [1.00-1.10]	0.069	1.03 [0.98-1.09]	0.274
	Sex	**2.30 [1.26-4.19]**	**0.006**	**3.27 [1.58-6.78]**	**0.001**	0.59 [0.28-1.24]	0.165	1.10 [0.42-2.88]	0.847
	ECOGPS[1]	1.40 [0.92-2.14]	0.120	1.25 [0.78-2.00]	0.346	**2.17 [1.05-4.49]**	**0.037**	1.98 [0.89-4.41]	0.096
	logSOD[2]	2.50 [0.81-7.77]	0.112	0.45 [0.10-1.95]	0.285	2.42 [0.18-32.77]	0.506	1.02 [0.06-17.89]	0.992
	logCA19-9	1.15 [0.92-1.42]	0.218	1.00 [0.81-1.24]	0.972	**0.70 [0.50-0.98]**	**0.038**	0.66 [0.42-1.04]	0.071

LoD = 0.10%		G12D N=50				G12V N=32			
		Univariate		Multivariate		Univariate		Multivariate	
	Variable	HR [95% CI]	p-value	HR [95% CI]	p-value	HR [95% CI]	p-value	HR [95% CI]	p-value
OS	logVAF	**1.71 [1.18-2.47]**	**0.004**	**2.22 [1.38-3.58]**	**0.001**	1.06 [0.66-1.69]	0.810	1.41 [0.79-2.53]	0.245
	Age	1.00 [0.97-1.03]	0.939	1.03 [0.99-1.07]	0.135	1.05 [1.00-1.09]	0.053	1.03 [0.97-1.09]	0.323
	Sex	**1.99 [1.08-3.67]**	**0.028**	**3.31 [1.50-7.30]**	**0.003**	**0.42 [0.20-0.92]**	**0.029**	0.50 [0.20-1.24]	0.135
	ECOGPS[1]	**1.77 [1.11-2.81]**	**0.016**	**1.70 [1.03-2.79]**	**0.037**	**1.89 [1.00-3.54]**	**0.049**	2.28 [0.98-5.30]	0.055
	logSOD[2]	2.67 [0.77-9.20]	0.121	0.48 [0.10-2.40]	0.375	2.95 [0.23-37.35]	0.404	0.23 [0.01-5.30]	0.355
	logCA19-9	1.15 [0.92-1.44]	0.231	1.03 [0.82-1.28]	0.825	0.80 [0.57-1.13]	0.206	0.92 [0.61-1.39]	0.690
PFS	logVAF	**1.68 [1.16-2.42]**	**0.006**	**2.15 [1.38-3.34]**	**0.001**	0.96 [0.64-1.45]	0.854	1.25 [0.77-2.03]	0.357
	Age	1.00 [0.97-1.03]	0.790	1.02 [0.98-1.05]	0.360	1.05 [1.00-1.10]	0.069	1.03 [0.98-1.09]	0.274
	Sex	**2.10 [1.14-3.87]**	**0.017**	**3.12 [1.49-6.57]**	**0.003**	0.59 [0.28-1.24]	0.165	1.10 [0.42-2.88]	0.847
	ECOGPS[1]	1.34 [0.86-2.09]	0.198	1.33 [0.81-2.17]	0.259	**2.17 [1.05-4.49]**	**0.037**	1.98 [0.89-4.41]	0.096
	logSOD[2]	1.99 [0.58-6.82]	0.273	0.46 [0.10-2.07]	0.313	2.42 [0.18-32.77]	0.506	1.02 [0.06-17.89]	0.992
	logCA19-9	1.11 [0.89-1.38]	0.374	1.00 [0.81-1.25]	0.973	**0.70 [0.50-0.98]**	**0.038**	0.66 [0.42-1.04]	0.071

LoD = 0.25%		G12D N=43				G12V N=30			
		Univariate		Multivariate		Univariate		Multivariate	
OS	logVAF	**1.68 [1.02-2.79]**	**0.042**	**2.08 [1.18-3.66]**	**0.011**	0.93 [0.53-1.64]	0.806	1.47 [0.71-3.03]	0.303
	Age	1.00 [0.97-1.03]	0.987	1.03 [0.99-1.07]	0.149	1.04 [0.99-1.09]	0.118	1.03 [0.97-1.09]	0.316
	Sex	**2.21 [1.14-4.29]**	**0.020**	**3.25 [1.40-7.51]**	**0.006**	0.47 [0.22-1.04]	0.061	0.50 [0.20-1.27]	0.146
	ECOGPS[1]	1.54 [0.82-2.89]	0.182	1.59 [0.84-3.01]	0.158	1.68 [0.88-3.20]	0.115	2.28 [0.93-5.58]	0.071
	logSOD[2]	1.45 [0.35-6.00]	0.605	0.38 [0.07-2.07]	0.262	2.69 [0.23-31.97]	0.433	0.19 [0.01-5.19]	0.328
	logCA19-9	1.08 [0.86-1.36]	0.493	1.02 [0.81-1.28]	0.882	0.80 [0.57-1.12]	0.201	0.96 [0.63-1.45]	0.840
PFS	logVAF	**1.76 [1.05-2.95]**	**0.031**	**1.98 [1.15-3.43]**	**0.014**	0.81 [0.50-1.32]	0.390	0.95 [0.48-1.88]	0.888
	Age	1.00 [0.96-1.03]	0.805	1.02 [0.98-1.06]	0.298	1.03 [0.98-1.09]	0.214	1.01 [0.95-1.08]	0.689
	Sex	**2.45 [1.26-4.76]**	**0.008**	**3.21 [1.42-7.27]**	**0.005**	0.70 [0.33-1.51]	0.366	1.45 [0.48-4.34]	0.511
	ECOGPS[1]	1.32 [0.71-2.44]	0.380	1.42 [0.75-2.67]	0.283	1.78 [0.85-3.71]	0.126	1.48 [0.59-3.70]	0.399
	logSOD[2]	1.58 [0.36-6.93]	0.543	0.48 [0.09-2.73]	0.410	2.12 [0.17-27.14]	0.564	1.53 [0.07-32.84]	0.787
	logCA19-9	1.06 [0.85-1.33]	0.597	0.99 [0.79-1.25]	0.957	**0.68 [0.48-0.96]**	**0.027**	**0.62 [0.38-1.02]**	**0.060**

[1]ECOG PS = Eastern Cooperative Oncology Group Performance Status

[2]SOD = Sum of diameters for all target lesions

Supplementary Table 5. Sensitivity analysis for LoDs (Limits of Detection) of 0.04% (as used for our assay, see Methods), 0.10%, and 0.25% for combined PRINCE and standard of care (SOC) cohorts. Shown in the top table are survival associations (hazard ratios, HR, with 95% confidence intervals (CI), and log-rank p-values) for baseline ctKRAS variant allele fraction (VAF) by variant (G12D on left and G12V on right) at the three LoDs. In the bottom table, Cox analysis is shown for baseline ctKRAS log of variant allele fraction (VAF) as a continuous variable for overall survival (OS) and progression-free survival (PFS), in each table. Significant values (without adjustment for multiple testing) indicated by bolded red text. Source data are provided as a Source Data file.

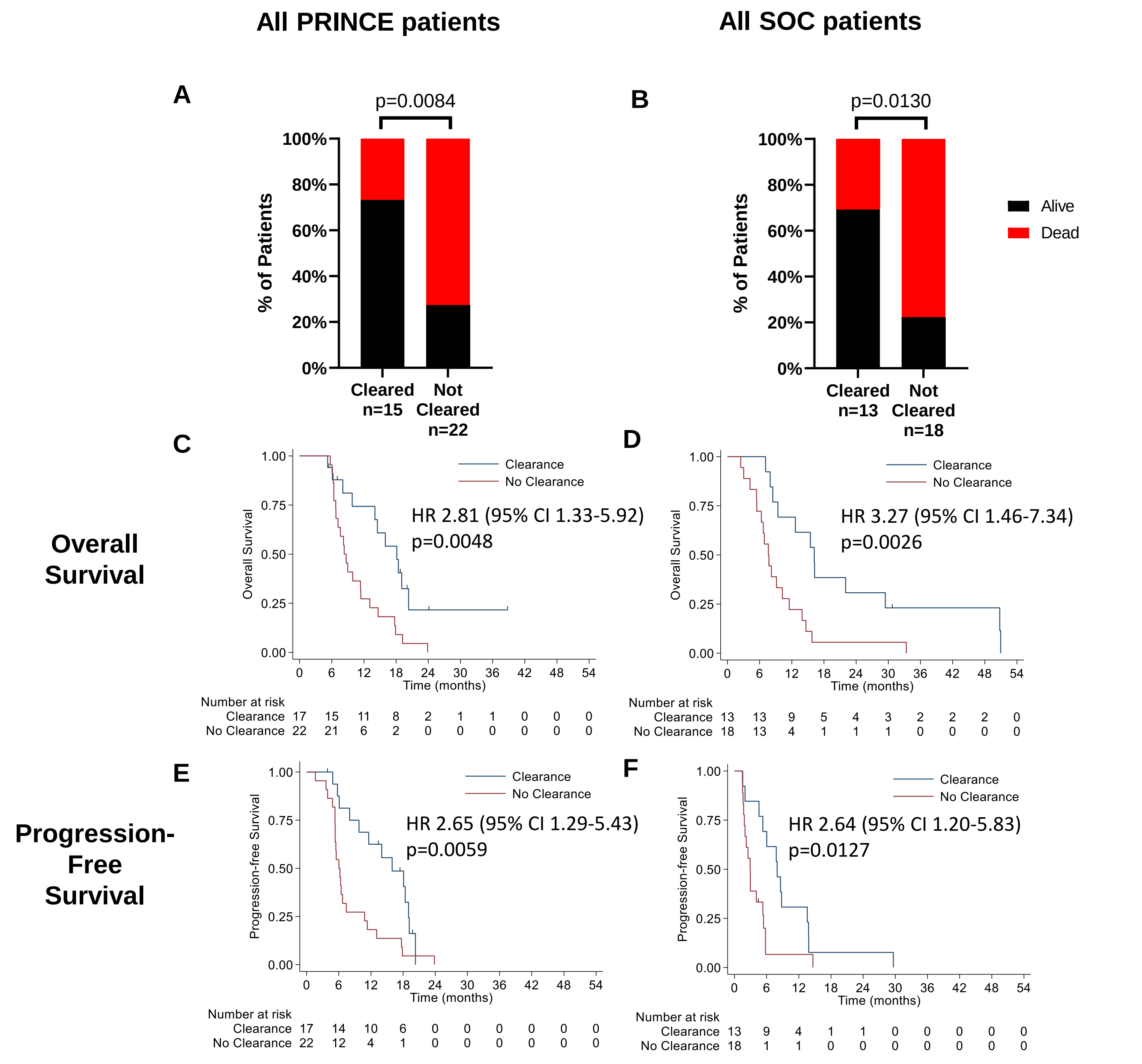

Supplementary Figure 9. ctKRAS clearance and association with survival for all therapy-naïve PRINCE and standard of care (SOC) patients with any ctKRAS variant detected. Includes (A, C, and E) all PRINCE patients, and (B, D, and F) SOC patients. Shown at top (A and B) is ctKRAS clearance at week 8 on therapy for all patients with one-year survival data (alive or dead at one year) (Fisher's Exact Test, two-sided), in the middle (C and D) is Kaplan-Meyer analysis for overall survival and on bottom (E and F) is progression-free survival. Cox regression hazard ratios (HR) and 95% confidence intervals (CI) are shown with log-rank p-values. Source data are provided as a Source Data file.

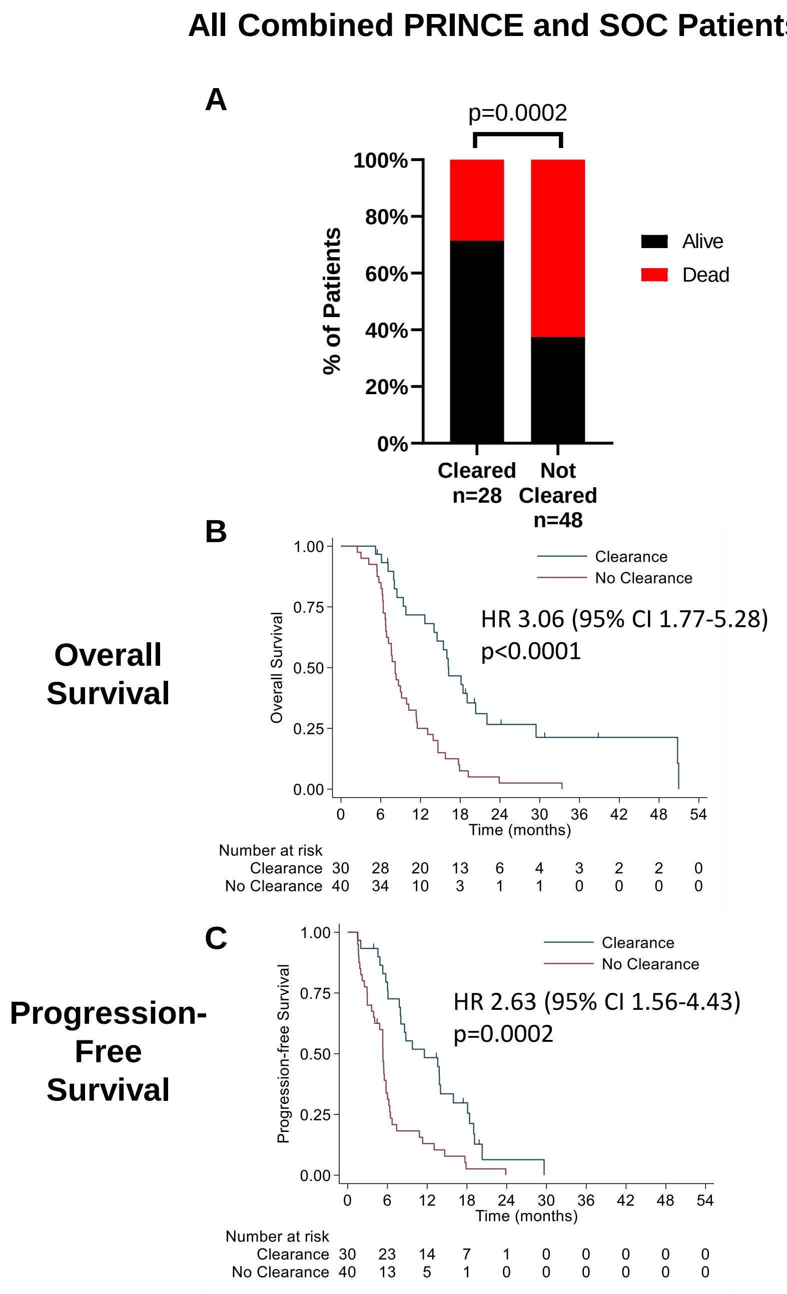

Supplementary Figure 10. ctKRAS clearance and association with survival for combined therapy-naïve PRINCE and standard of care (SOC) patients with any ctKRAS variant detected. Shown at top (A) is ctKRAS clearance at week 8 on therapy for all patients with one-year survival data (alive or dead at one year) (Fisher's Exact Test, two-sided), in the middle (B) is Kaplan-Meyer analysis for overall survival and on bottom (C) is progression-free survival. Cox regression hazard ratios (HR) and 95% confidence intervals (CI) are shown with log-rank p-values. Source data are provided as a Source Data file.

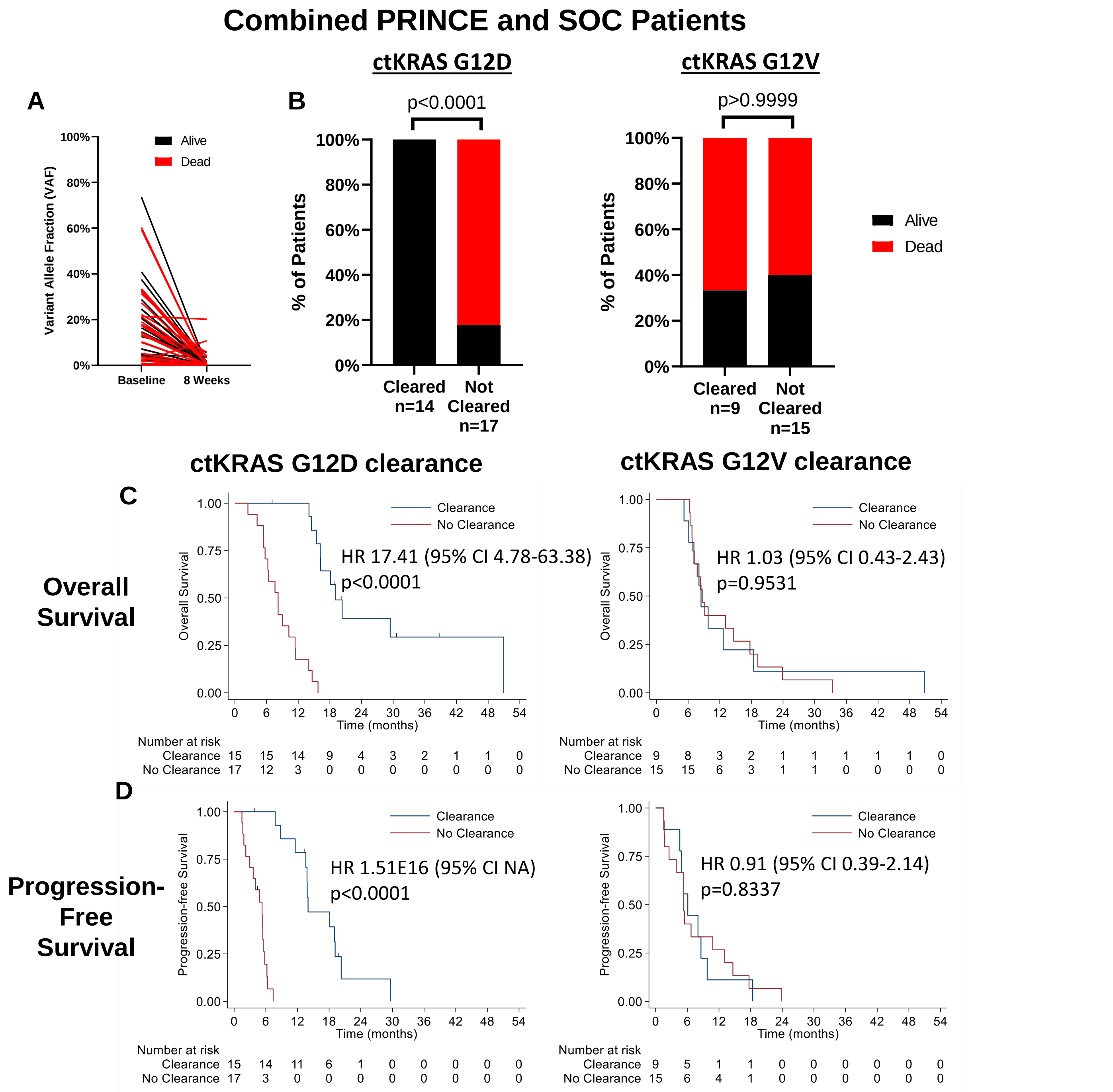

Supplementary Figure 11. Survival association with early, on-therapy ctKRAS dynamics by variant for combined therapy-naïve PRINCE and standard of care (SOC) cohort. Shown is the association with one-year survival (alive or dead at one year) for the combined PRINCE and SOC cohorts with any detected baseline ctKRAS mutation as measured by (A) changes in ctKRAS variant allele fraction (VAF) from baseline to week 8 on therapy (n=37), or (B) ctKRAS clearance at week 8 on therapy for G12D only (left) and G12V only (right). Shown in (C) is Kaplan-Meyer analysis dichotomized by ctKRAS clearance vs no clearance and association with overall survival for G12D only (left), and G12V only (right). Results for progression-free survival shown in D. Among the 39 PRINCE patients included in the combined cohort with both a baseline and week 8 plasma obtained, 2 had insufficient follow-up to determine 1-year survival and were thus excluded from the results shown in A and B. Mann-Whitney test used for comparisons in A. Fishers exact test (two-sided) used in B. Cox regression hazard ratios (HR) and 95% confidence intervals (CI) are shown with log-rank p-values for C and D. Source data are provided as a Source Data file.

	PRINCE						SOC					
	G12D			G12V			G12D			G12V		
	ρ	p	n	ρ	p	n	ρ	p	n	ρ	p	n
SOD-All lesions	**0.4160**	**0.0160**	**33**	0.0633	0.7743	23	**0.6182**	**0.0003**	**30**	0.3497	0.2662	12
SOD-Metastases	**0.3830**	**0.0278**	**33**	0.3386	0.1140	23	**0.6384**	**0.0001**	**30**	0.1831	0.5693	12
SOD-Pancreas lesions	0.2096	0.2417	33	-0.1672	0.4458	23	0.0798	0.6588	33	0.1652	0.5124	18
COL-All lesions	**0.4878**	**0.0040**	**33**	0.3459	0.1060	23	**0.4927**	**0.0057**	**30**	0.3929	0.2062	12
COL-Metastases	**0.5011**	**0.0030**	**33**	0.3459	0.1060	23	**0.4927**	**0.0057**	**30**	0.4008	0.1964	12
COL-Pancreas lesions	-0.0886	0.6241	33	NA	NA	23	-0.2229	0.2125	33	-0.2572	0.3028	18
CA19-9	0.2246	0.2700	26	-0.0675	0.7712	21	**0.4171**	**0.0196**	**31**	0.1272	0.6037	19
Albumin	-0.2911	0.1003	33	-0.3967	0.0609	23	**-0.3831**	**0.0253**	**34**	-0.3826	0.0959	20
ALT	0.1500	0.4048	33	**0.4839**	**0.0193**	**23**	0.2655	0.1291	34	0.3237	0.1639	20
AST	**0.3641**	**0.0372**	**33**	**0.6695**	**0.0005**	**23**	0.3142	0.0703	34	0.391	0.0883	20
BUN	0.0099	0.9564	33	-0.1281	0.5603	23	0.0748	0.6743	34	0.2166	0.359	20
Creatinine	-0.1637	0.3626	33	-0.2936	0.1739	23	0.2225	0.2060	34	0.1204	0.6131	20
Bilirubin	0.1221	0.4983	33	0.2762	0.2021	23	0.0507	0.7757	34	0.3851	0.0936	20
Age	-0.1038	0.5655	33	-0.1706	0.4365	23	-0.0940	0.5970	34	-0.2918	0.2118	20

Supplementary Table 6. Spearman correlation analysis (two-sided) of clinical variables and ctKRAS VAF levels for therapy-naïve PRINCE and SOC patients. SOD=Sum of diameters, COL=Count of lesions. Significant values indicated by bolded red text. NA indicates variable without variation in the cohort. Abbreviation ALT (Alanine Transaminase), AST (Aspartate Transaminase), and BUN (Blood Urea Nitrogen) are used. Source data are provided as a Source Data file.

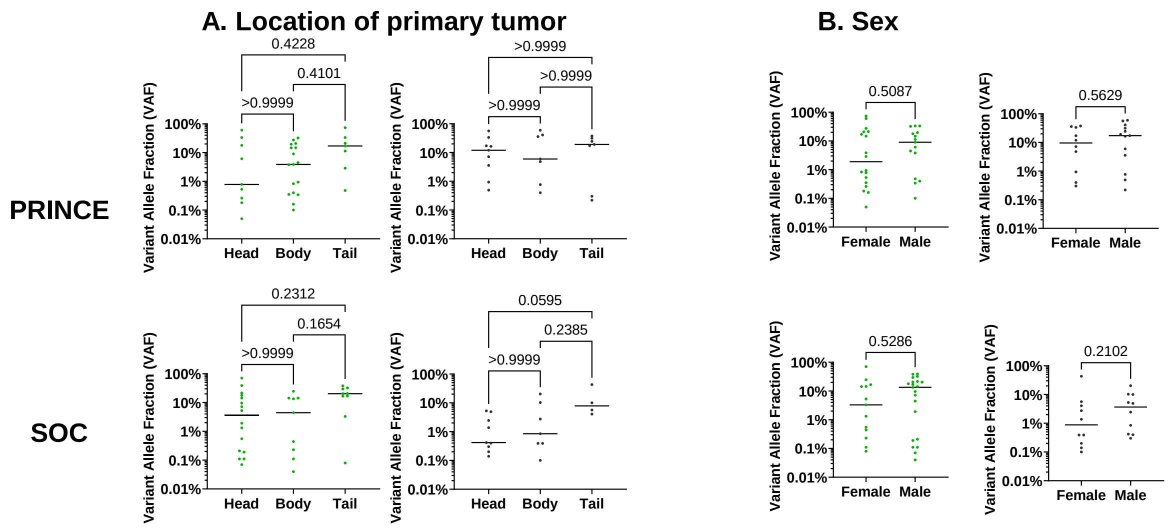

Supplementary Figure 12. Association of primary tumor location and sex with baseline ctKRAS VAF levels for therapy-naïve PRINCE and standard of care (SOC) patients. Plots demonstrating association between baseline ctKRAS VAF levels and (A) primary tumor location (Dunn's multiple comparisons test) or (B) sex (Mann-Whitney test, two-sided). Asterisk indicates significance at 0.05. Green denotes ctKRAS G12D and dark grey is G12V. Source data are provided as a Source Data file.
