# Supplementary material for: Circulating KRAS G12D but not G12V is associated with survival in metastatic pancreatic ductal adenocarcinoma
Source: Nat Commun. 2024 Jul 9;15:5763. doi: 10.1038/s41467-024-49915-5 (PMC11233636; doi:10.1038/s41467-024-49915-5)
Supplement: Supplementary file 3 — Reporting Summary [file 41467_2024_49915_MOESM3_ESM.pdf]

nature portfolio

Corresponding author(s): Erica L. Carpenter

Last updated by author(s): May 21, 2024

Reporting Summary

Nature Portfolio wishes to improve the reproducibility of the work that we publish. This form provides structure for consistency and transparency in reporting. For further information on Nature Portfolio policies, see our Editorial Policies and the Editorial Policy Checklist.

Statistics

For all statistical analyses, confirm that the following items are present in the figure legend, table legend, main text, or Methods section.

n/a	Confirmed	
☐	☒	The exact sample size (*n*) for each experimental group/condition, given as a discrete number and unit of measurement
☐	☒	A statement on whether measurements were taken from distinct samples or whether the same sample was measured repeatedly
☐	☒	The statistical test(s) used AND whether they are one- or two-sided<br>*Only common tests should be described solely by name; describe more complex techniques in the Methods section.*
☐	☒	A description of all covariates tested
☐	☒	A description of any assumptions or corrections, such as tests of normality and adjustment for multiple comparisons
☐	☒	A full description of the statistical parameters including central tendency (e.g. means) or other basic estimates (e.g. regression coefficient) AND variation (e.g. standard deviation) or associated estimates of uncertainty (e.g. confidence intervals)
☐	☒	For null hypothesis testing, the test statistic (e.g. *F*, *t*, *r*) with confidence intervals, effect sizes, degrees of freedom and *P* value noted<br>*Give P values as exact values whenever suitable.*
☒	☐	For Bayesian analysis, information on the choice of priors and Markov chain Monte Carlo settings
☒	☐	For hierarchical and complex designs, identification of the appropriate level for tests and full reporting of outcomes
☒	☐	Estimates of effect sizes (e.g. Cohen's *d*, Pearson's *r*), indicating how they were calculated

Our web collection on statistics for biologists contains articles on many of the points above.

Software and code

Policy information about availability of computer code

Data collection	PRINCE clinical data was collected in Medidata Rave EDC.<br>Droplet digital PCR analysis was performed on either the RainDrop system (RainDance Techologies) or QX200 AutoDG System (Bio-Rad).<br>For whole exome and transcriptome sequencing, FFPE tumor samples were profiled<br>using ImmunoID NeXT (Personalis, Inc., Menlo Park, CA, USA), an augmented exome/transcriptome<br>platform and analysis pipeline. Paired-end sequencing was performed on NovaSeq instrumentation<br>(Illumina, San Diego, CA, USA).
Data analysis	Kaplan-Meier, log-rank, and Cox regression analyses were performed in Stata/IC 16.1 (Stata Corp.). Spearman correlation, Dunn's multiple comparisons, Fisher's exact, Chi-square, and two-tailed Mann-Whitney analyses were performed in Prism 9.5.1 (GraphPad Software). Droplet Digital PCR data were analyzed using RainDrop Analyst version 1.1.0 (RainDance Technologies) or QuantaSoft Version 1.7.4 (Bio-Rad). Plots of the estimated restricted cubic spline function relating single predictors to survival were generated in R (version 4.2.1) using the survival, rms, and ggplots2 packages and combining various functions to, e.g., compute restricted cubic splines, and fit a Cox proportional hazards regression model.

For manuscripts utilizing custom algorithms or software that are central to the research but not yet described in published literature, software must be made available to editors and reviewers. We strongly encourage code deposition in a community repository (e.g. GitHub). See the Nature Portfolio guidelines for submitting code & software for further information.

Data

Policy information about availability of data

All manuscripts must include a data availability statement. This statement should provide the following information, where applicable:

- Accession codes, unique identifiers, or web links for publicly available datasets
- A description of any restrictions on data availability
- For clinical datasets or third party data, please ensure that the statement adheres to our policy

Genetic data and clinical data are available in the supplemental files. Confidential or identifiable patient information, subject to patient privacy, cannot be shared.

Research involving human participants, their data, or biological material

Policy information about studies with human participants or human data. See also policy information about sex, gender (identity/presentation), and sexual orientation and race, ethnicity and racism.

Reporting on sex and gender	The manuscript includes self-reported sex data as recorded in the Medical Record for PennMedicine Standard of Care (SOC) patients and as collected as part of the clinical trial data at participating sites for the PRINCE patients. Gender data was not available at the time of data collection and thus not included. Individual sex data is provided in the source data file. The PRINCE cohort contained 35 female subject and 48 male subjects and the SOC cohort contained 37 female subjects and 48 males subjects.<br>Sex was only considered in determining if there was a difference between the two cohorts, which there was not (Fishers Exact test P = 0.8773). Sex was also considered in the univariate and multivariate Cox analyses (Supplemental Tables 4 and 5).
Reporting on race, ethnicity, or other socially relevant groupings	The manuscript includes race and ethnicity data as recorded in the Medical Record for PennMedicine Standard of Care (SOC) patients and as collected as part of the clinical trial data at participating sites for the PRINCE patients. Reported races were categorized as “Asian,” “Black or African American,” “Caucasian,” or “Other.” Ethnicities were categorized as either “Hispanic or Latino” or “Not Hispanic or Latino.”<br>Race and ethnicity were only considered in determining if there was a difference between the two cohorts, which there was not (race, Chi-square test, P = 0.1089; ethnicity, Fishers Exact test P = 0.5529). Further race or ethnicity-based analysis as confounding variables was not possible due to insufficient numbers.
Population characteristics	For the PRINCE clinical trial we refer you to the initial publication in Nature Medicine, Padron et al, (https://doi.org/10.1038/s41591-022-01829-9).<br>For the PennMedicine SOC cohort, patient characteristics are outlined in Supplementary Table 2.
Recruitment	For the PRINCE clinical trial we refer you to the initial publication in Nature Medicine, Padron et al, (https://doi.org/10.1038/s41591-022-01829-9)<br>PennMedicine SOC patients were selected by research staff based on study criteria pending approval by treating oncologist and informed consent.<br>We are not aware of any biases that may be present that may have impacted our results.
Ethics oversight	The PRINCE clinical trial (PICI0002, NCT03214250) was approved by the lead (University of Pennsylvania) institutional review board and accepted at all participating sites. It was conducted in compliance with the Declaration of Helsinki and International Conference on Harmonisation Good Clinical Practice guidelines. Written informed consent was provided by all 129 patients with mPDAC prior to enrollment. For more details we refer you to the initial publication in Nature Medicine, Padron et al, (https://doi.org/10.1038/s41591-022-01829-9).<br>For the PennMedicine SOC cohort, collection protocol and all amendments were approved by the Institutional Review Board at the University of Pennsylvania. The study was conducted in accordance with the Declaration of Helsinki.

Note that full information on the approval of the study protocol must also be provided in the manuscript.

Field-specific reporting

Please select the one below that is the best fit for your research. If you are not sure, read the appropriate sections before making your selection.

☒ Life sciences ☐ Behavioural & social sciences ☐ Ecological, evolutionary & environmental sciences

For a reference copy of the document with all sections, see nature.com/documents/nr-reporting-summary-flat.pdf

Life sciences study design

All studies must disclose on these points even when the disclosure is negative.

Sample size	Sample size for the PRINCE clinical trial was determined based on the trial endpoint, this is unrelated to the analysis presented here for which all available data was considered as described in the manuscript. Sample size for the PennMedicine SOC cohort was determined by total accrual at the time the study assay was performed, no power calculation was used and no preset sample size was set.
Data exclusions	For the PRINCE clinical trial cohort, overall, ctKRAS variants were detected for 86 of 115 (74.8%) available patients. For the 19 PRINCE trial patients with detectable ctKRAS who had received therapy prior to trial enrollment, ctKRAS variant allele fraction (VAF) was significantly lower

	than for the 67 patients with de novo stage IV disease (p<0.0001, Supplemental Figure 1); therefore, patients with prior treatment were excluded from further analysis. Additionally, four PRINCE patients had both a G12D and a G12V KRAS variant detected in plasma and these patients were excluded from analysis in which variant-specific differences in OS or PFS were calculated. For the multivariate Cox regression analysis CA19-9 was excluded from PRINCE and SOD excluded from SOC cohort due to incomplete data. For combined PRINCE and SOC analysis of the 33 and 31 patients analyzed in the PRINCE and SOC tables respectively for G12D, only the 53 patients with both CA19-9 and SOD values available were included in combined cohort.For the correlative analysis presented in Figure 6, exact numbers are presented in Supplemental Table 5 as appropriate imaging and clinical variables were not available for all patients.
Replication	The initial findings of variant specific ctDNA associations with survival in the PRINCE clinical trial cohort were verified in the independent PennMedicine SOC cohort. Per reviewer comments and editorial instructions, these results were also verified in the combination of the two cohorts. Further, exploratory correlative analysis between tumor metrics or clinical values and ctKRAS variant allele fraction for the PRINCE cohort were also investigated in the Penn Medicine SOC cohort for all patients with available imaging and clinical data.
Randomization	his study was observational in nature, comparing genetically defined groups (tumor bearing mutation based). Allocation was not random with respect to the comparisons made in the present study. Covariates are outlined in Supplemental Table 4. Within each cohort, there were no statistically significant differences in patient characteristics between patients with KRAS G12D- versus G12V-bearing tumors.
Blinding	Researchers performing ctDNA assays were blinded to sample outcomes, blinding to genetic group was not possible as assays were group specific. Blinding is not relevant as the analyses were post-hoc and exploratory.

Reporting for specific materials, systems and methods

We require information from authors about some types of materials, experimental systems and methods used in many studies. Here, indicate whether each material, system or method listed is relevant to your study. If you are not sure if a list item applies to your research, read the appropriate section before selecting a response.

Materials & experimental systems

n/a	Involved in the study
☒	☐ Antibodies
☒	☐ Eukaryotic cell lines
☒	☐ Palaeontology and archaeology
☒	☐ Animals and other organisms
☐	☒ Clinical data
☒	☐ Dual use research of concern
☒	☐ Plants

Methods

n/a	Involved in the study
☒	☐ ChIP-seq
☒	☐ Flow cytometry
☒	☐ MRI-based neuroimaging

Clinical data

Policy information about clinical studies

All manuscripts should comply with the ICMJE guidelines for publication of clinical research and a completed CONSORT checklist must be included with all submissions.

Clinical trial registration	NCT03214250
Study protocol	The phase 1b/2 PRINCE study (PICI0002, NCT03214250) was approved by lead (University of Pennsylvania) institutional review board and accepted at all participating sites. It was conducted in compliance with the Declaration of Helsinki and International Conference on Harmonisation Good Clinical Practice guidelines. Written informed consent was provided by all 129 patients with mPDAC prior to enrollment. PRINCE clinical trial protocol is available in the Padron et al Nature Medicine publication (https://doi.org/10.1038/s41591-022-01829-9).<br>Patients in the Penn Medicine SOC cohort were enrolled under IRB Protocol #822028 (attached with submission) after obtaining written informed consent. The study was conducted in accordance with the Declaration of Helsinki.
Data collection	For the PRINCE clinical trial we refer you to the initial publication in Nature Medicine, Padron et al, (https://doi.org/10.1038/s41591-022-01829-9).<br>For the PennMedicine SOC cohort samples were collected from August 14, 2015 through September 9, 2018. Clinical data was collected from the medical records between August 14, 2015 and June 29, 2021.
Outcomes	This study was observational, post-hoc, and exploratory in nature. There were no predefined end-points.

Plants

Seed stocks	*Report on the source of all seed stocks or other plant material used. If applicable, state the seed stock centre and catalogue number. If plant specimens were collected from the field, describe the collection location, date and sampling procedures.*
Novel plant genotypes	*Describe the methods by which all novel plant genotypes were produced. This includes those generated by transgenic approaches, gene editing, chemical/radiation-based mutagenesis and hybridization. For transgenic lines, describe the transformation method, the number of independent lines analyzed and the generation upon which experiments were performed. For gene-edited lines, describe the editor used, the endogenous sequence targeted for editing, the targeting guide RNA sequence (if applicable) and how the editor was applied.*
Authentication	*Describe any authentication procedures for each seed stock used or novel genotype generated. Describe any experiments used to assess the effect of a mutation and, where applicable, how potential secondary effects (e.g. second site T-DNA insertions, mosiacism, off-target gene editing) were examined.*
